# Comparison between skeletal muscle and adipose tissue measurements with high-dose CT and low-dose attenuation correction CT of ^18^F-FDG PET/CT in elderly Hodgkin lymphoma patients: a two-centre validation

**DOI:** 10.1259/bjr.20200672

**Published:** 2021-06-09

**Authors:** Domenico Albano, Luca Camoni, Roberto Rinaldi, Alessandra Tucci, Vittorio Ruggero Zilioli, Cristina Muzi, Marco Ravanelli, Davide Farina, Alessandra Coppola, Manuela Camalori, Raffaele Giubbini, Francesco Bertagna

**Affiliations:** 1 Nuclear Medicine, University of Brescia and ASST Spedali Civili Brescia, Brescia, Italy; 2 Nuclear Medicine, ASST Spedali Civili Brescia, Brescia, Italy; 3 Hematology Division, ASST Spedali Civili Brescia, Brescia, Italy; 4 Haematology, ASST Grande Ospedale Metropolitano Niguarda, Milan, Italy; 5 Department of Radiology, University of Brescia, Brescia, Italy; 6 Department of Radiology, Grande Ospedale Metropolitano Niguarda, Milan, Italy

## Abstract

**Objectives:**

High-dose CT (HDCT) is considered the gold-standard imaging for the measurements of skeletal muscle area (SMA), visceral adipose tissue (VAT), subcutaneous adipose tissue (SAT) and intramuscular adipose tissue (IMAT) areas in the abdomen. These parameters may reflect sarcopenia, which can have a prognostic impact in several oncological diseases. The aim of this study was to compare the agreement of measurements of SMA, VAT, SAT and IMAT areas between HDCT and low-dose CT (LDCT) of 18-fludeoxyglucose positron emission tomography (^18^F-FDG PET)/CT in elderly patients affected by Hodgkin lymphoma (HL).

**Methods:**

We retrospectively included 90 patients affected by HL who underwent baseline ^18^F-FDG-PET/CT and HDCT within a mean interval of 7 days. HDCT and LDCT images were analysed by two blinded observers using segmentation software (Slice-O-Matic, Tomovision) to quantify the areas. HDCT and LDCT measurements were compared using Bland–Altman plots and Passing-Bablock regression analyses. Pearson correlation coefficient (r) was used to correlate measurements from the two imaging modalities.

**Results:**

Comparison of HDCT and LDCT data demonstrated a strong correlation for measurement of VAT(*r* = 0.942, *p* < 0.0001), SAT (*r* = 0.894, *p* < 0.0001) and SMA (*r* = 0.934, *p* < 0.0001). Instead considering IMAT, correlation was good but less significant (*r* = 0.742). The mean difference between the two methods was found to be very small, with a difference of 1% for SAT,+6.1% for VAT,+2.5% for SMA and −1.9% for IMAT.

**Conclusion:**

LDCT of PET/CT is a safe, accurate and precise method for the measurements of skeletal muscle area, visceral and subcutaneous adipose tissue. Their measurements are reproducible and correlate closely with HDCT.

**Advances in knowledge:**

LLDCT of PET/CT is a safe and accurate method for the measurements of SMA, VAT and SAT; their measurements are closely correlated with HDCT. LDCT can be considered an accurate alternative tool for measuring abdominal fat and muscles in the clinical practice.

## Introduction

Sarcopaenia is defined as the loss of the skeletal muscle mass and function and it is mainly a phenomenon age-related.^1^ With ageing, a reduction in the muscle fibre size and number is physiological and causes a loss of the muscle mass of approximately 30%.^2,3^ The causes of sarcopaenia are multifactorial and potentially associated with different conditions, such as endocrine dysfunctions, muscle disuses, gender, chronic diseases (like inflammation and nutritional deficiencies) or iatrogenic.^4^ The clinical meaning of the sarcopaenia is widely recognised, but its definition and radiological assessment method are still under discussion. Early attempts to define the sarcopaenia were based on the measurements of the skeletal muscle mass with dual energy X-ray absorptiometry (DXA) in relation to the body size, but they presented significant limitations; *e.g.* DXA is unable to evaluate the intramuscular fat, which can account for 5–15% of observed muscle mass.^5^ High-dose CT (HDCT) is a tool frequently used in oncological patients for the staging or restaging purposes and it may be used to assess the total and fat-free muscle area, with a smaller risk of error than that compared to DXA.^6^ MRI has a similar accuracy and reproducibility for the fat and muscle measurements and may be used also for a whole-body imaging study. Both CT and MRI can be able to discriminate tissue compartments in the abdomen with a good accuracy.^7–12^ The segmentation analysis of a single axial CT image at the third lumbar vertebra (L3) is a reference method for the body composition assessment, particularly in the oncologic setting.^1^ The advantages of CT are its wide availability, its relative low cost and its speed. For these reasons, CT is recognised as one of most reliable methods for the *in-vivo* quantification of the body composition,^13,14^ while MRI has been increasingly adopted in this field due to radiation exposure concerns as well as the potential to achieve improved tissue contrast.

The clinical and prognostic role of the sarcopaenia has already been demonstrated in several oncologic diseases and an important clinical impact was also reported for lymphoma patients.^15–21^ However, studies based on Hodgkin lymphoma (HL) are so far rare.

Fluorine-18-fludeoxyglucose positron emission tomography/CT (^18^F-FDG PET/CT) is a non-invasive tool with proven usefulness in the evaluation of HL in the staging, treatment response evaluation and prognosis.^22,23^ There are also several studies showing the important role of ^18^F-FDG-PET/CT in elderly patients.^24,25^

The CT part of a PET/CT system is not always a contrast-enhanced HDCT due to the fact that the main aims are to provide precise anatomical localisation of radiotracer uptake identified on the PET images and the attenuation correction of the PET emission data. To reach this target also a low-dose CT (LDCT) may be sufficient and some centres opt for this system to reduce radiation exposure to the patients.

However, no studies to our knowledge have compared the measurements of the abdominal skeletal muscle area and adipose tissue areas between these two modalities (HDCT and LDCT of PET)

The purpose of this bicentric retrospective study was to investigate the agreement and correlation between HDCT and LDCT of PET measurements of the adipose tissue and skeletal muscle area in elderly HL.

## Methods and materials

### Patients

In this retrospective study, 90 patients with newly diagnosed histologically proven HL were selected consecutively from the institutional electronic patient health record of two tertiary care centres between January 2010 and December 2019 (66 patients from one centre and the remaining 24 form the other). The main features of our population are summarised in Table 1. All patients performed both a whole body ^18^F-FDG PET/CT and a high-dose thoracic-abdominal CT within a maximum interval of 10 days (median 5, range 1–10 days).

**Table 1.** Baseline features of our population

	Patients n (%)
Age years mean ± SD (range)	72.2 ± 5 (65–85)
Sex male	45 (50%)
Sex female	45 (50%)
Tumour stage at diagnosis (Ann Arbor)	
I	4 (4%)
II	21 (24%)
III	30 (33%)
IV	35 (39%)
BMI > 25	34 (38%)
B symptoms	43 (48%)
Bulky disease	9 (10%)
LDH ≤245	49 (54%)
>245	41 (46%)
Bulky disease	9 (10%)
Histotype: classic	59 (66%)

BMI, Body mass index; HDCT, High-dose CT; IMAT, Intramuscular adipose tissue; LDCT, Low-dose CT; LDH, Lactate dehydrogenase; SAT, Subcutaneous adipose tissue; SD, Standard deviation; SMA, Skeletal muscle area; VAT, Visceral adipose tissue.

### ^18^F-FDG PET/CT imaging

The ^18^F-FDG PET/CT acquisition was performed according to the standard operating procedures,^26^ after at least 6 h fasting and with blood glucose levels < 150 mg dl^−1^. An activity of 3.5 MBq/kg was administered intravenously (mean activity injected 245 MBq, range 161–315 MBq) and the imaging was acquired 60 min after injection from the skull base to the mid-thigh (2.5 min per bed; steps of 15 cm), using a Discovery ST-E (D-STE) or 690 (D-690) scanner (General Electric, Milwaukee, WI) or on a Biograph True Point PET/CT tomograph (Siemens Medical Solutions, Erlangen, Germany). For all scanners, a standard non-contrast free-breathing helical LDCT was obtained for morphologic correlation and attenuation correction. The D-STE acquisition parameters were: 120 kV, fixed tube current ~73  mAs (40–160 mAs), eight slices × 3.75 mm and 3.27 mm interval, pitch 1:5, tube rotation 0.8 s. The D-690 and Biograph True Point acquisition parameters were: 120kV, fixed tube current ~60  mAs (40–100 mAs), 64 slices × 3.75 mm and 3.27 mm interval, pitch 0.984:1, tube rotation 0.5 s. The PET/CT images were reconstructed using a 512 × 512 matrix and iterative reconstruction, 3.75 mm slice thickness and 3.25 mm interval, standard filter with a window setting with 400 Hounsfiled units (HUs) of window width and 40 HU of window level. Patients were instructed to void before imaging acquisition, no oral or intravenous contrast agents were administrated or bowel preparation used for any patient; written consent was obtained before studies.

### CT imaging

HDCT scans were performed on 15 different scanners of 4 different CT manufacturers: 5 General Electric scanners (a Brightspeed, a Lightspeed Plus, a Lightspeed VCT, a Optima CT520 series, and a Optima CT660), 5 Siemens scanners (a Somatom Definition Flash, a Sensation 16 and a Scope), 2 Toshiba scanner (a Aquilion and a Asteion), and 2 Philips scanner (a Brilliance 64 and a Brilliance 16). The acquisition parameters were: tube voltage settings were selected 100, 120 and 130 kV and tube current in a range of values from 65 to 389 mAs (mean 150). The CT images were reconstructed using a 512 × 512 matrix and standard filter. Contrast enhancements venous phase images were used for the measurements in 35 examinations, while in the remaining 55 cases not-enhanced images were selected. The main technical features are resumed in Supplementary Table 1.

Supplementary Table 1.

### Imaging analysis

HDCT and LDCT images were analysed by two researchers (DA, AC) for the measurements of the adipose and muscular tissues using Slice-O-Matic software v. 4.2 (Montreal, Quebec, Canada Tomovision). Each researcher analysed the images of scans performed in his centre.

An axial section with a multiplanar reconstruction at third lumbar vertebra was used to measure the skeletal muscle area (SMA) considering psoas, paraspinal, abdominal transverse rectum, internal and external obliques and visceral, subcutaneous and intramuscular adipose tissue (VAT, SAT, IMAT). CT HU thresholds were –29 to 150 for SMA, –190 to –30 for SAT, –190 to –30 IMAT, and –50 to –150 for VAT. The tissue margins were manually corrected as needed.

A second observer (RR) of one centre made the muscular and adipose areas measurements randomly in 45 patients (both HDCT and LDCT scans) already evaluated by another researcher in order to calculate the interobserver variability.

### Statistical analysis

All statistical analysis was carried out using MedCalc Software v. 17.1 for Windows (Ostend, Belgium). The numeric variables were described as mean, minimum and maximum. The descriptive analysis of the categorical variables comprised the calculation of the simple and relative frequencies.

The intraclass correlation coefficient (ICC) was calculated for interobserver agreement of HDCT and LDCT measurements of SMA, VAT, SAT and IMAT; this analysis was performed by two blinded observers in 45 patients.

The Bland–Altman analysis was performed to plot the mean difference percentage (bias) between HDCT and LDCT measurements of SMA, SAT, VAT and IMAT. The agreement between the two imaging modalities were calculated by the Passing-Bablok regression analysis.

## Results

Among the 45 patients evaluated by 2 operators, the HDCT measurements of all body parameters (SMA, VAT, SAT, IMAT) were very similar between the two observers (Table 2); also using LDCT these evidences were confirmed (ICC higher than 0.900 in all cases).

**Table 2.** Interobserver agreement for the evaluation of SMA, VAT, SAT and IMAT in 45 patients

HDCT	Observer 1	Observer 2	ICC
SMA (cm^2^) mean ± SD	112.4 ± 29	114.9 ± 30.3	0.936
VAT (cm^2^) mean ± SD	145 ± 85	139.9 ± 74	0.929
SAT (cm^2^) mean ± SD	149.9 ± 63.2	150.7 ± 65	0.940
IMAT (cm^2^) mean ± SD	16.8 ± 10.6	15.4 ± 10.2	0.924
LDCT			
SMA (cm^2^) mean ± SD	113.2 ± 25.5	114.2 ± 23.9	0.959
VAT (cm^2^) mean ± SD	139.4 ± 84.2	140.9 ± 85.5	0.955
SAT (cm^2^) mean ± SD	144.5 ± 71	146 ± 72.2	0.933
IMAT (cm^2^) mean ± SD	18.9 ± 13.8	17.4 ± 12.8	0.909

HDCT, High-dose computed tomography; ICC, Intraclass coefficient correlation; IMAT, Intramuscul aradipose tissue; LDCT, Low-dose computed tomography; SAT, Subcutaneous adipose tissue; SMA, Skeletal muscle area; VAT, Visceral adipose tissue.

**Figure 1.**
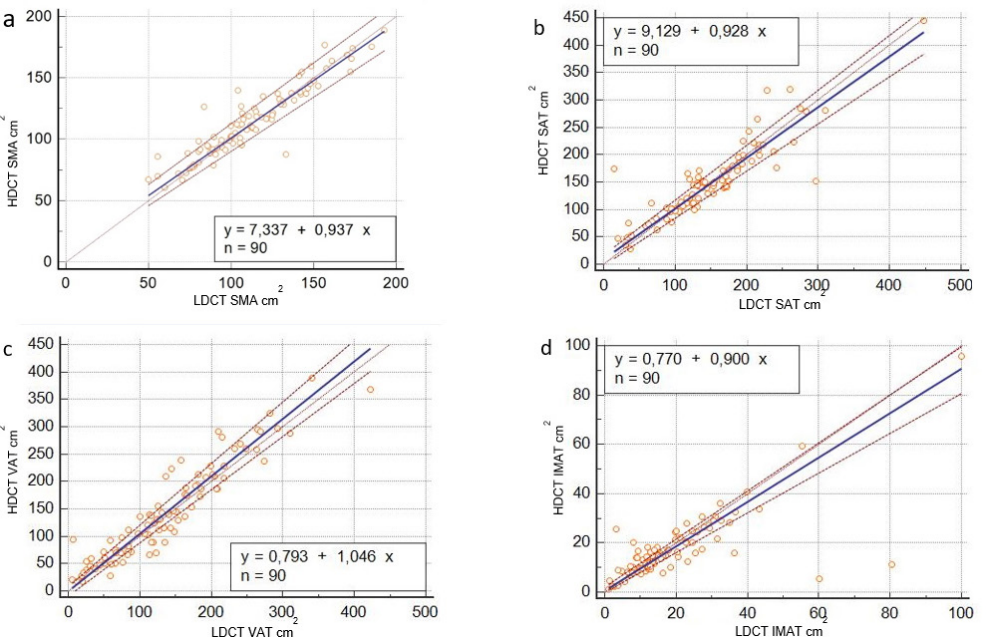
Comparison between HDCT and LDCT in measuring SMA (**a**), SAT (**b**), VAT (**c**) and IMAT (**d**) using Passing-Bablok analysis (blue lines represent Passing Bablock fit; red lines 95% confidence intervals bands). HDCT, high-dose computedtomography; IMAT, intramuscular adiposetissue; LDCT, low-dose CT; SAT, subcutaneous adiposetissue; SMA, skeletal muscle area; VAT, visceral adipose tissue.

**Figure 2.**
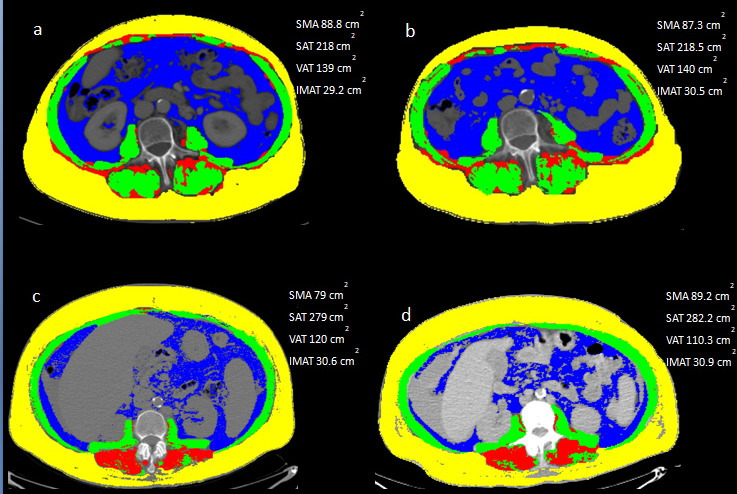
A representative case of measurements of SAT (yellow), VAT (blue), IMAT (red) and SMA (green) using HDCT (**a**) and LDCT (**b**) images. Another example with the comparison between HDCT (**c**) and LCT (**d**). HDCT, high-dose computedtomography; IMAT, intramuscular adiposetissue; LDCT, low-dose CT; SAT, subcutaneous adiposetissue; SMA, skeletal muscle area; VAT, visceral adipose tissue.

Applying the Passing-Bablock regression analysis, the comparison between HDCT and LDCT data (Figure 1) demonstrated a good agreement for the measurements of SMA (intercept of 7.337, 95% CI −1.805 to 13.547; slope of 0.937, 95% CI 0.883 to 0.991; *r* = 0.919, *p* < 0.0001), VAT (intercept of 0.793 95% CI −9.315 to 9.001; slope of 1.045, 95% CI 0.974 to 1.124; *r* = 0.941, *p* < 0.0001) and SAT (intercept of 9.129, 95% CI −2.439 to 17.314; slope of 0.927, 95% CI 0.862 to 1.001; *r* = 0.901, *p* < 0.0001) and a moderate agreement of IMAT (intercept of 0.769, 95% CI −0.203 to 2.306; slope of 0.900, 95% CI 0.809 to 0.971; *r* = 0.749, p 0.001) (Table 3). Cusum test for linearity indicates no significant deviation from linearity for SMA (*p* = 0.94), VAT (*p* = 0.46), SAT (*p* = 0.46) and IMAT (*p* = 0.46). Some representative examples are reported in Figure 2.

**Table 3.** correlation analysis between HDCT and LDCT (*n* = 90)

	All population (n 90)			
	HDCT	LDCT	Correlation coefficient	*p*-value
SMA (cm^2^) mean ± SD (range)	114.4 ± 29 (60-188)	111.9 ± 31 (49.9–192)	0.919	<0.001
VAT (cm^2^) mean ± SD (range)	143.4 ± 87 (17.1–368)	137.3.4±83 (5-422)	0.941	<0.001
SAT (cm^2^) mean ± SD (range)	149.9 ± 67.9 (47–444)	149 ± 71.3 (14.5–448)	0.901	<0.001
IMAT (cm^2^) mean ± SD (range)	17.1 ± 12.6 (1.2–95.7)	19 ± 15.6 (1–99)	0.749	<0.001
	**Centre n°1 (n 66**)			
	**HDCT**	**LDCT**	**Correlation coefficient**	***p*** **-value**
SMA (cm^2^) mean ± SD (range)	113.9 ± 27.6 (66.8–188)	115 ± 28.8 (68–192)	0.971	<0.001
VAT (cm^2^) mean ± SD (range)	142.3 ± 86.8 (17.1–368)	139.8 ± 83.9 (5–422)	0.942	<0.001
SAT (cm^2^) mean ± SD (range)	155.1 ± 67.5 (47–444)	155.6 ± 71.7 (14.5–448)	0.945	<0.001
IMAT (cm^2^) mean ± SD (range)	17.8 ± 14.1 (1.2–95.7)	20.5 ± 17.4 (1–99)	0.939	<0.001
	**Center n°2 (n 24**)			
	**HDCT**	**LDCT**	**Correlation coefficient**	***p*** **value**
SMA (cm^2^) mean ± SD (range)	115.8 ± 33.3 (60–176.8)	103.8 ± 35.8 (49.9–170)	0.888	<0.001
VAT (cm^2^) mean ± SD (range)	146.2 ± 89.7 (17.2–389)	130.7 ± 82.6 (11.9–341)	0.967	<0.001
SAT (cm^2^) mean ± SD (range)	136.3 ± 68.5 (27.8–317)	131.4 ± 68.4 (30.5–297)	0.911	<0.001
IMAT (cm^2^) mean ± SD (range)	15.1 ± 7.2 (2.5–31.7)	15.1 ± 8.6 (3.6–33.4)	0.570	0.004

HDCT, High-dose computed tomography; IMAT, intramuscular adipose tissue; LDCT, Low-dose CT; SAT, Subcutaneous adipose tissue; SMA, Skeletal muscle area; VAT, Visceral adipose tissue.

The Passing-Bablock regression analysis for each center demonstrated similar results with the exception of IMAT, where the measurements were significantly different (Table 3).

A high agreement with LDCT was found for both contrast-enhanced and unenhanced HDCT (VAT *r* = 0.934, SAT *r* = 0.910, SMA *r* = 0.930 considering contrast-enhanced scans; VAT *r* = 0.950, SAT *r* = 0.878, SMA *r* = 0.938 considering unenhanced scans, respectively).

**Figure 3.**
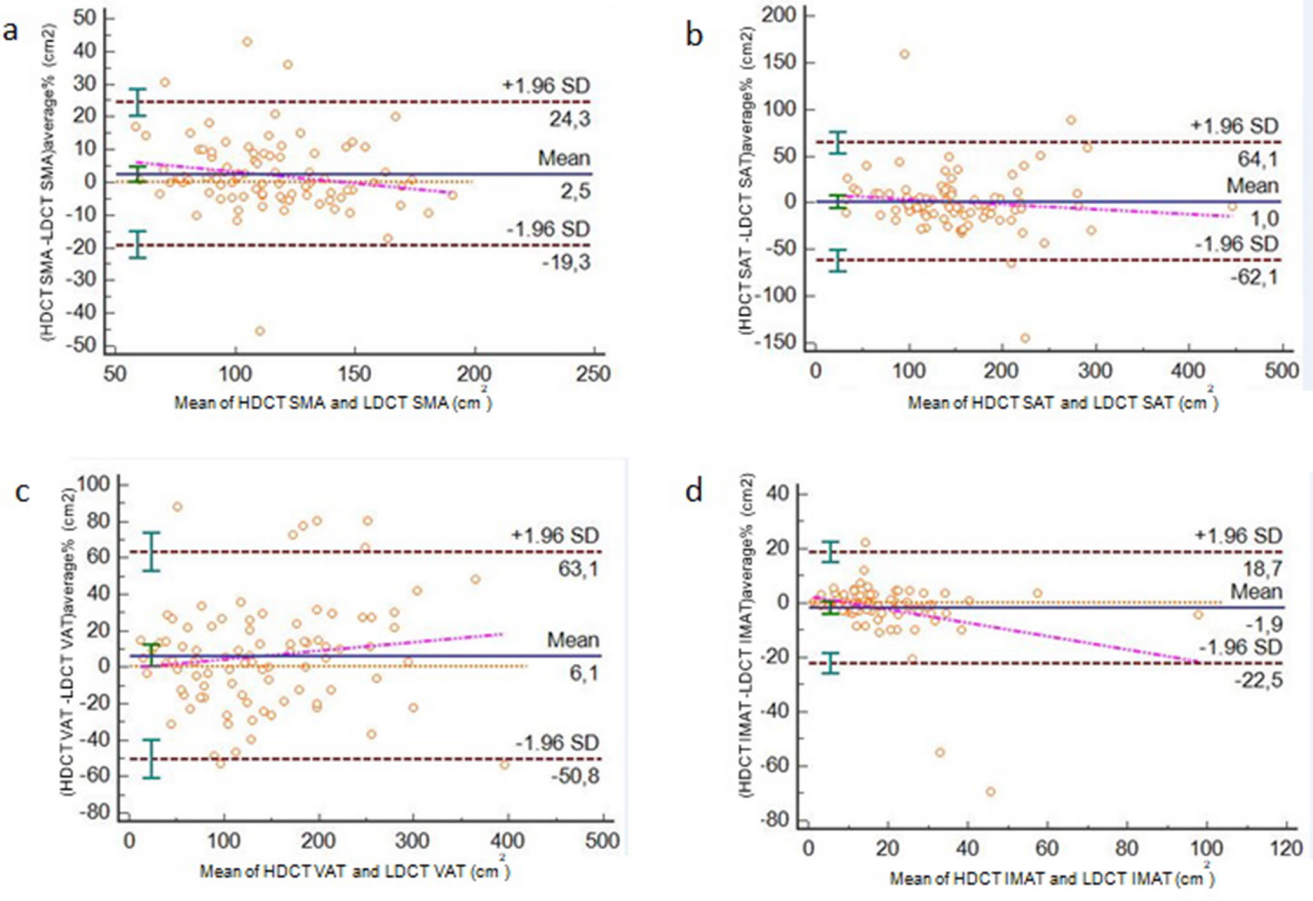
Bland–Altman plots which compare difference in HDCT and LDCT for SMA (**a**), SAT (**b**), VAT (**c**) and IMAT (**d**) (blue lines represent mean; red lines 95% limits; orange lines bias ; violet lines the trend). HDCT, high-dose computedtomography; IMAT, intramuscular adiposetissue; LDCT, low-dose CT; SAT, subcutaneous adiposetissue; SMA, skeletal muscle area; VAT, visceral adipose tissue.

Applying Bland–Altman analysis, the mean difference between the two methods was small (Figure 3), with a value of 2.5% for SMA, +6,1% for VAT, 1% for SAT and −1.9% for IMAT but with wide limits of agreement especially for VAT (limits of agreement −50.8% to +63.1%) e SAT (limits of agreement −62.1% to +64.31%) and moderate for SMA (limits of agreement −19.3% to +24,3%) and IMAT (limits of agreement −22.5% to +18.7%).

## Discussion

Our analysis showed a good inter- and intraobserver agreement for the measurements of SMA, VAT and SAT between the HDCT and LDCT images; only for IMAT both the inter- and the intraobserver agreement were less significant and reproducible. Moreover, a significant difference between the two centres in IMAT measurements was registered, while for other factors the agreement was good. These evidences are well showed considering Figure 1 and Table 3; for IMAT measurements there were some cases markedly different between HDCT and LDCT. This discrepancy may be explained by the low area of adipose tissue present usually between muscular fibres (average value lower than SAT and VAT). Therefore, also a minimal difference (as a potential error operator-dependent) in the measurements may be expressed as a statistically significant difference. Moreover, the detection and the measurement of IMAT is technically challenging in comparison with the other parameters especially due to the difficulties to recognize safely the muscular margins. For this reason, among our parameters, we may certainly support the idea that the IMAT analysis is the most difficult and less reproducible. For the other variables (SAT, VAT, SMA), a less number of discordant cases was described, except of one patient where there was a significantly high difference in the SAT evaluation probably related to an operator-dependent error.

Passing–Bablok regression analysis showed no systematic differences and no proportional differences for all parameters (for SMA intercept of 7.337, 95% CI −1.805 to 13.547; slope of 0.937, 95% CI 0.883 to 0.991; for VAT intercept of 0.793 95% CI −9.315 to 9.001; slope of 1.045, 95% CI 0.974 to 1.124; for SAT intercept of 9.129, 95% CI −2.439 to 17.314; slope of 0.927, 95% CI 0.862 to 1.001), except of proportional difference for IMAT (intercept of 0.769, 95% CI −0.203 to 2.306; slope of 0.900, 95% CI 0.809 to 0.971). Applying Passing Bablok regression analysis, we demonstrated that the constant and proportional bias between the two methods were not significant with the exception of IMAT.

Also applying the Bland–Altman analysis, that is a statistical method utilised in the assessment of difference in measurement techniques, we found a very small difference considering the mean value between the body estimates utilising HDCT and LDCT, ranging from −1,9 to 6%. Instead observing 95% limits of agreement for VAT and SAT comparison, the differences were high (>60%), indicating that large intervals in measurements were present. The effective clinical meaning of these wide intervals needs to be clarify and could be potentially affected by the relatively low sample analysed. This evidence reduce the effective positive agreement derived by Bland–Altman analysis. Instead for IMAT and SMA, the differences were lower despite the 95% limits of agreement remained quite high underlying that the two methods were relatively equivalent for the measurement of these factors.

Thus, these preliminary results reflect the potential reproducibility of these two techniques suggesting a possible role of LDCT in estimating the muscular and adipose areas, despite the high agreement and reproducibility was not achieved.

A crucial point derived from our study, is that the abdominal muscular and adipose tissue areas can be measured with high accuracy also by LDCT of PET/CT. CT of PET/CT may be a LDCT because usually doesn't have strictly a diagnostic role. Its main roles are the attenuation correction and the anatomical localisation, which are crucial for the evaluation and quantification of PET images and improve the diagnostic performance.^27^

Another potential usefulness to study the accuracy of LDCT in measuring these parameters is the situation when the patients performed HDCT in a different centre compared to PET/CT scan with the risk that electronic images of one these two examinations are not always available due to the local policies and/or organisational issues. In this case, the measurements of muscular and adipose areas could be done with the tool available.

The potential impact of the contrast enhancement in the measurements of the body parameters is not yet clear with only few reports present in literature but with controversial results. Boutin et al^28^ demonstrated that the contrast injection influenced the skeletal muscle and bone changing attenuation value, but they also showed that the contrast enhancement may vary significantly with age, gender, and unenhanced tissue attenuation and, in the case of muscle, by anatomic region.

In our analysis, we preferred to use unenhanced images of HDCT for the comparison of the muscular and adipose areas with LDCT if possible (*n* = 55) and we utilised the contrast-enhanced venous phase HDCT images as alternative (*n* = 35), but we derived a high correlation for both kind of images. This evidence underlies the concept that the contrast enhancement could not affect significantly this kind of measurements.

Other authors found an excellent agreement between non-contrast and contrast SMA measurements^29^ or a not clinically relevant differences in skeletal muscle mass measurements.^30^ In contrast, Feng et al^31^ observed a significant difference between the unenhanced and arterial and venous phases. In our analysis, we didn't find any differences using unenhanced and enhanced phases of HDCT; the correlation and the agreement with LDCT is not significantly different and is high in all cases. Instead for the subcutaneous or visceral adipose tissues, no comparison between not-enhanced and enhanced images are available.

Unlike other papers that included heterogeneous populations,^28–31^ one of the strength of our paper is the fact that we studied a specific population: all elderly patients affected by HL.

For the study of HL, ^18^F-FDG PET/CT has a crucial role both in the staging and evaluation of treatment response fields.^22^ Recent evidences have suggested a possible clinical and prognostic role of sarcopaenia in lymphoma,^15–21^ especially in elderly patients. We can intrinsically consider SMA as a part of the diagnostic definition of sarcopaenia, then its measurement appears crucial and we need a feasible and validated method. ^18^F-FDG PET/CT is usually used for the evaluation of metabolic behaviour and FDG-avidity of lymphoma; adding also a body composition analysis (particularly SAT, VAT and SMA) can make this evaluation more complete and solid.

Nowadays, the body composition analysis (like the measurements of muscular and adipose areas) is not routinely diffuse in clinical practice due to the not shared clinical meaning and the lack of time-efﬁcient and clinical-friendly assessment tools that could allow accurate muscle and adipose tissue measurements. The analysis of CT images requires specific segmentation of different tissue areas which can be not easy. Several semi-automated software for the analysis of body composition from medical imaging are available, like Slice O-Matic, but most of these methods have not been externally evaluated in large, real-world data sets.

For this reason, the research of an automated system to estimate the body composition can be crucial with the aim to reduce the workload and to accelerate the research in the body composition and chronic disease outcomes by leveraging the vast repositories of imaging data available within health systems.^32^

The demonstration of a good accuracy and the validation of LDCT in the measurements of the adopenic and muscular areas as a surrogate of sarcopaenia may have several clinical advantages such as the possibility to have both metabolic and morphological information from the same tool (PET/CT), the reduced radiation exposure and the immediacy of sarcopaenic measurements after scan.

Despite further studies are needed to confirm or controvert our findings, these measurements may help to change early the patient’s management or better stratify the clinical features of the patients.

The limitations of our study are the retrospective nature of the study design, the heterogeneity of CT scanner and protocols used (with or without enahnced-contrast) and the relatively low number of patients analysed, also due to the rarity of the disease. Another limitation is the interobserver agreement evaluated only in half of the population included. Despite this, so far, the present study represents the first series of elderly HL in which HDCT and LDCT were compared in the measurement of the skeletal muscles and adipose tissues.

In conclusion, LDCT of PET/CT is a safe, accurate and precise method for the measurements of the skeletal muscle area, visceral and subcutaneous adipose tissue. Their measurements are reproducible and correlate closely with HDCT imaging. Compared with HDCT, LDCT is a possible accurate alternative for measuring abdominal fat and muscles in clinical practice.
