# Supplementary material for: Comparison between skeletal muscle and adipose tissue measurements with high-dose CT and low-dose attenuation correction CT of 18F-FDG PET/CT in elderly Hodgkin lymphoma patients: a two-centre validation
Source: Br J Radiol. 2021 Jun 9;94(1123):20200672. doi: 10.1259/bjr.20200672 (PMC8248212; doi:10.1259/bjr.20200672)
Supplement: Supplementary Table 1. [file bjr.20200672.suppl-01.doc]

**SUPPLEMENTAL TABLE 1: CT scanner model and features**

| **CT scan model** | **kV** | **mAs mean (range)** | **ATCM system** |
| --- | --- | --- | --- |
| GE Brightspeed (n 1) | 120 | 205 | Smart mA |
| GE Lightspeed Plus (n 1) | 120 | 171 | Smart mA |
| GE Lightspeed VCT (n 1) | 120 | 300 | Smart mA |
| GE Optima CT520 series (n 4) | 120 | 276 (226-319) | Smart mA |
| GE Optima CT660 (n 3) | 100/120 | 202 (200-204) | Smart mA |
| Philips Brilliance 64 (n 27) | 120 | 185 (77-389) | I-Dose |
| Philips Brilliance 16 (n 2) | 120 | 232 (204-260) | I-Dose |
| Siemens Somatom Definition Flash (n 5) | 120 | 175 (141-232) | Care Dose 4D |
| Siemens Somatom Definition 64 (n 7) | 120 | 126 (73-197) | Care Dose 4D |
| Siemens Sensation 16 (n 13) | 100/120 | 178 (91-328) | Care Dose 4D |
| Siemens Somatom Volume Zoom (n 1) | 120 | 140 | Care Dose 4D |
| Siemens Somatom Sensation 64 (n 12) | 120 | 108 (81-144) | Care Dose 4D |
| Siemens Scope (n 1) | 130 | 99 | Care Dose 4D |
| Toshiba Asteion (n 1) | 120 | 75 | Sure Exposure |
| Toshiba Aquilon (n 11) | 120 | 169 (65-360) | Sure Exposure |
